# The Impact of Systemic Oncological Treatments on the Fertility of Adolescents and Young Adults—A Systematic Review

**DOI:** 10.3390/life13051209

**Published:** 2023-05-18

**Authors:** Justine Himpe, Sander Lammerant, Lore Van den Bergh, Lore Lapeire, Chloë De Roo

**Affiliations:** 1 Department of Medical Oncology, Ghent University Hospital, 9000 Ghent, Belgium; 2 Department of Internal Medicine and Pediatrics, Faculty of Medicine and Health Sciences, Ghent University, 9000 Ghent, Belgium; 3 Faculty of Medicine and Health Sciences, Ghent University, 9000 Ghent, Belgium; 4 AYA Research Centre and Hub (ARCH), Ghent University, 9000 Ghent, Belgium; 5 Department of Reproductive Medicine, Ghent University Hospital, 9000 Ghent, Belgium

**Keywords:** oncofertility, gonadotoxicity, reproductive health, chemotherapy, targeted therapy, immunotherapy, monoclonal antibodies, tyrosine kinase inhibitors, adolescents and young adults, AYA

## Abstract

Background: Over the past decades, advancements in oncological treatments have led to major improvements in survival. Particularly for adolescents and young adults (AYAs), fertility is an important concern in cancer survivorship. The purpose of the review is to provide physicians with a practical overview of the current knowledge about the impact of systemic oncological treatments on the fertility of female and male AYAs. Methods: A systematic review was performed based on relevant articles obtained from 4 databases up until 31 December 2022. Results: The mechanisms of gonadotoxicity and the concurrent risk is described for the following categories: chemotherapy, targeted therapy and immunotherapy. For the category “chemotherapy”, the specific effects and risks are listed for the different classes and individual chemotherapeutics. In the category “targeted therapy”, a distinction was made between tyrosine kinase inhibitors (TKIs) and monoclonal antibodies. Information concerning immunotherapy is scarce. Conclusions: The effects of chemotherapy on fertility are well investigated, but even in this category, results can be conflicting. Insufficient data are available on the fertility effects of targeted therapy and immunotherapy to draw definitive conclusions. More research is needed for these therapies and their evolving role in treating cancers in AYAs. It would be useful to include fertility endpoints in clinical trials that evaluate new and existing oncological treatments.

## 1. Introduction

Cancer in adolescents and young adults (AYAs) is defined by the National Cancer Institute and by the working group of the European Society for Medical Oncology (ESMO) and the European Society for Paediatric Oncology (SIOPE) as diagnoses occurring among those aged between 15 and 39 years [1,2]. In 2019, 1,335,100 new malignancies were diagnosed in AYA patients worldwide, with an incidence of 45 per 100,000 people [3]. The most common malignancies in AYAs (>90% of cases) are leukaemias, lymphomas, sarcomas, melanoma, breast cancer, testicular cancer, colorectal cancer, thyroid cancer and brain tumours. In the last few years, there has been increasing awareness of the specific needs of AYAs with cancer. One of those needs is fertility, which is also depicted as one of the thirteen key challenges in AYA cancer care proposed by the ESMO-SIOPE working group [1]. 

As treatments for malignancies become increasingly successful and survival rates for many types of cancer improve, although, at a slower pace in AYAs, more and more cancer patients become cancer survivors [4]. Specifically for AYAs, fertility is an important part of the quality of life in cancer survivorship [5,6]. Possible long-term adverse effects of standard cancer treatments such as surgery, radiotherapy and systemic therapy on fertility are temporary or permanent impaired gonadal function and infertility. 

The field of systemic cancer therapy is continuously evolving, and next to classic cytotoxic chemotherapy, targeted therapy and immunotherapy have become increasingly incorporated into the standard of care. The impact of chemotherapeutic agents on fertility is best described in the literature, comprising varying degrees of gonadotoxicity depending on the mechanism of action, dose and length of treatment, and age of the patient at the time of treatment. For other systemic cancer treatments, the harmful effects on fertility are less known [7,8,9].

Due to gonadotoxic treatment, primary ovarian insufficiency (POI) can occur as a long-term effect of anti-cancer treatment. POI is defined as the loss of normal ovarian function before a woman reaches the age of 40. It is characterized by secondary amenorrhea due to hypergonadotropic hypogonadism. Other symptoms of POI are caused by estrogen deficiency, for example, hot flashes, sweating at night, mood swings/disorders, sleep disorders, lack of concentration, arthralgia and reduced sexual function. It is also associated with loss of bone mineral density and a higher risk of cardiovascular events [10].

Patients should be well-informed about gonadal function and fertility before and after treatment and offered different strategies for fertility and fertility preservation [7,8,11,12]. In this regard, physicians need to be educated about reproductive health, the effects of systemic therapies on fertility and quality of life, and fertility preservation strategies. Therefore, the purpose of the review is to provide the physician with a practical overview of the current knowledge about the impact of systemic oncological treatments on the fertility of female and male adolescents and young adults. 

## 2. Materials and Methods

This systemic review is based on a literature search in 4 databases, including PubMed, Embase, Google Scholar and Web of Science. Articles published in English until 31 December 2022 were considered. This manuscript also includes articles from decades ago because of their lasting relevance up to now. 

The population of interest consists of adolescents and young adults, defined in this review as ‘people between 15 and 39 years old at cancer diagnosis’. Therefore, all articles about patients of this age were included. Articles in which some patients did not fit these specific age limitations, but others did not, were also included. When the age was not specifically mentioned, but it could be assumed from the wording that the article was about AYAs, these articles were also included. When selecting specific types of treatment and treatment schedules, special attention was given to treatments for cancer types specific to the above-mentioned population. Regarding immunotherapy, this review focuses on immune checkpoint inhibitors because these are widely used in current clinical practice compared with other treatments, such as CAR-T cell therapy, tumour-infiltrating lymphocytes or cancer vaccines. Animal studies were excluded. Preclinical studies were only considered when focussing on human patients. Review articles were only included if they contained extra relevant information compared with the original articles.

The search terms were ‘chemotherapy/targeted therapy/immunotherapy/monoclonal antibodies/tyrosine kinase inhibitors’ and/or ‘cancer’ and/or ‘cancer treatment’ and/or ‘(onco)fertility’ and/or ‘guidelines’ and/or ‘reproductive health’ and/or ‘gonadotoxic effects’ and/or ‘gonadal function (evaluation)’ and/or ‘semen analysis’ and/or ‘fertility damage’ and/or ‘side effects’ and/or ‘gonadotoxic impact’ and/or ‘adolescents and young adults/AYA’. 

**Figure 1:**
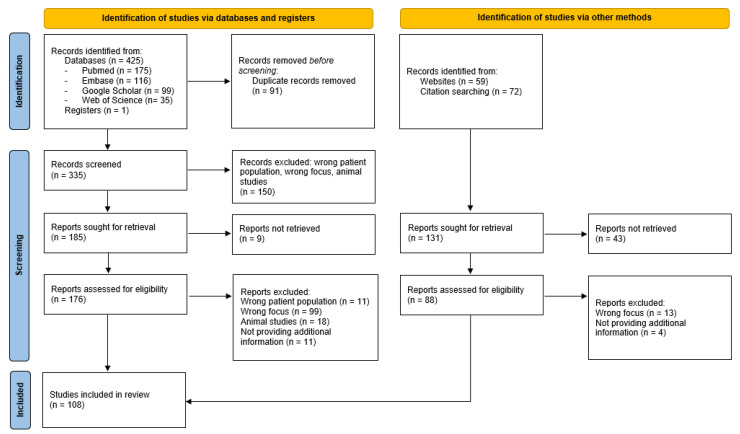
PRISMA 2020 flow diagram for systematic reviews.

Articles that fit the aforementioned criteria were considered for inclusion. If an article did not provide the right focus, it was excluded. The article search is illustrated in the PRISMA flowchart (Figure 1). The review was performed by 3 researchers. 

This review is registered in the PROSPERO registry under number 409256.

## 3. Results

### 3.1. Women

#### 3.1.1. Folliculogenesis and Measuring Gonadal Function

At birth, the ovaries have about 1 to 2 million oocytes forming the ovarian reserve, of which only 300 will reach maturity during reproductive life. The oocyte count progressively decreases until menopause [13,14]. The follicles are the functional unit of the ovaries where the oocyte matures and is surrounded by granulosa and theca cells [14]. There are two major phases of folliculogenesis: the preantral period, which is gonadotropin-sensitive, and the antral period, which is gonadotropin-dependent and occurs after puberty. In the antral stage, most follicles undergo apoptosis. Only a few continue to grow to the preovulatory stage [15]. 

The ovarian reserve determines a woman’s fertility potential, both for spontaneous conception and pregnancy, through assisted reproduction techniques. Several markers are used to estimate ovarian reserve, such as anti-Müllerian hormone (AMH) levels [13,16] and antral follicle count (AFC) [13]. Other markers for evaluation of ovarian function include the incidence of amenorrhea, gonadotropin levels and estradiol levels. AMH is a hormone secreted by granulosa cells and is involved in regulating primordial follicle recruitment. The main source of AMH is the pool of preantral and small antral follicles present in the ovaries. The AMH blood level represents the ovarian follicular pool and is a useful quantitative marker of ovarian reserve [17]. There may be a correlation between AMH levels before gonadotoxic treatment and subsequent ovarian reserve, but the relation to reproductive capacity after treatment remains controversial [13]. The AFC is measured by transvaginal ultrasound in the early follicular phase [17]. Furthermore, the incidence of amenorrhea is often reported as a surrogate marker for ovarian reserve [17]. FSH can also be used as a marker of gonadal function, as it is elevated when there is a loss of ovarian function. Preferably, FSH is measured at the beginning of the menstrual cycle in the early follicular phase [17]. At last, LH and estradiol can also be used to monitor ovarian function and reserve [18].

In general, gonadotoxicity is expressed as the risk of developing permanent amenorrhea. It is divided into three classes: high risk, intermediate risk and low to very low risk, with a chance of permanent amenorrhea of more than 80%, 20–80% and less than 20%, respectively [13]. 

#### 3.1.2. Mechanisms of Gonadotoxicity

##### Chemotherapy

The impact of chemotherapy on fertility has been known and reported since the 1970s [19]. More recent studies found that women are 13–38% less likely to become pregnant after a cancer diagnosis and treatment with chemotherapy compared with the general population [20,21]. Chemotherapy can lead to early irreversible oocyte depletion, varying from a reduced number of follicles to complete absence. The degree of ovarian damage greatly varies depending on drug exposure (type of drug and cumulative dose of chemotherapy), gonadal reserve before treatment and patient age [22,23]. Determining the ovarian impairment of individual antitumor drugs is difficult as, in practice, most chemotherapy regimens consist of multiple drugs [24].

**Figure 2:**
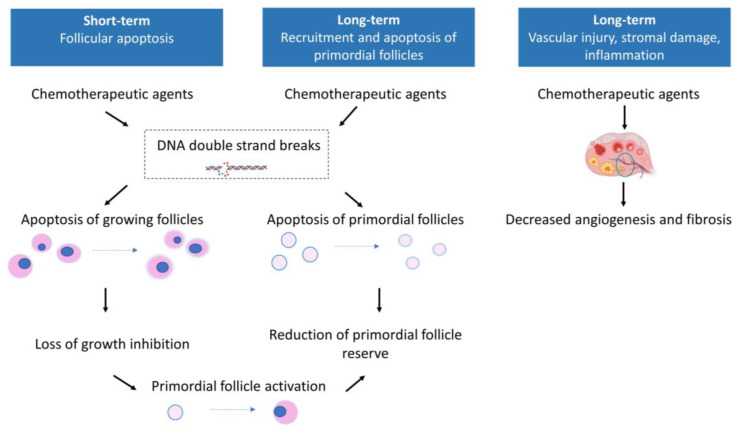
Mechanisms of gonadotoxicity of chemotherapy in women.

Chemotherapeutic agents act through different mechanisms in the ovary (Figure 2). Some act in a cell-cycle-dependent way (for example, antimetabolites), while others are not dependent on cell division (for example, alkylating agents, anthracyclines and platinum agents). Both oocyte and granulosa cells can be damaged by chemotherapy, and there are hypotheses that even the primordial follicles are targeted [17]. This impact on the ovaries can be distinguished into short-term and long-term effects. 

The short-term effects are responsible for the loss of growing follicles and cause amenorrhea soon after the beginning of chemotherapy [15,17,23,25,26]. Short-term effects are caused by DNA alterations in oocytes and/or mitotic cells and stromal injury. First, double-stranded breaks are the most severe DNA lesions caused by cytotoxic agents resulting in follicle death by apoptosis. For example, alkylating agents and anthracyclines induce double-stranded breaks in the DNA of growing follicles [17]. Platinum-based compounds bind to DNA and form DNA cross-links leading to DNA breakage during replication and, eventually, follicular apoptosis [17,27]. Taxanes inhibit microtubule formation and spindle function in mature oocytes, affecting short-term reproductive potential [27]. Antimetabolites cause inhibition of DNA, RNA, thymidylate and purine synthesis of growing follicles, but they do not affect primordial follicles [17,28]. Anthracyclines are reported to induce oxidative stress in oocytes, resulting in mitochondrial dysfunction and further cellular damage [24,29]. Second, all classes of chemotherapeutic agents target mitotic cells, such as granulosa cells, and can alter follicle development of growing follicles [17]. Third, both alkylating and nonalkylating drugs affect ovarian stromal function, leading to a substantial decrease in estradiol production [24]. 

The long-term effects are responsible for the loss of ovarian reserve and subsequent POI and infertility [15]. Chemotherapy treatment can result in significant loss of ovarian reserve, and because the ovarian reserve decreases with age, the risk of POI increases in older women [13,17,18,25,29,30,31,32,33]. Goodwin et al. reported a 15–30% chance of POI in breast cancer patients under 35 years of age compared with 50% in women above 40 years old [34]. An important remark regarding ovarian ageing is that not only the quantity of oocytes decreases with age but also the oocyte quality [23]. Long-term effects are caused by permanent damage to the ovaries through primordial follicle death or depletion and vascular injury, fibrosis or stromal damage [18,29]. 

First, the loss of primordial follicles can be caused by direct DNA alterations or by accelerated recruitment. For alkylating agents and anthracyclines, DNA alterations and double-stranded breaks can lead to apoptosis of primordial follicles. Although, some follicles will be able to repair DNA damage and survive. In the case of platinum-based compounds, there seems to be no direct toxicity in primordial follicles [17]. Another existing hypothesis for primordial follicle depletion suggests that chemotherapy (such as alkylating agents and platinum-based compounds) accelerates the recruitment of dormant follicles in an attempt to replace the damaged follicles, causing a “burn-out” effect [10,13,17]. Primordial follicles develop into more fragile primary or secondary follicles, giving the ovaries a second hit when further chemotherapy treatment is administered [35]. Recruitment of primordial follicles would be induced by the activation of the phosphoinositide 3-kinase (PI3K) signalling pathway, whose role in follicle quiescence has been well-established. This pathway is activated in response to a reduction of AMH secretion following follicle apoptosis [15]. Interestingly, some chemotherapeutic agents that induce the death of growing follicles, for example, antimetabolites and, therefore, loss of inhibition of follicle recruitment, are not shown to deplete ovarian reserve [29]. 

Second, vascular injury, fibrosis and stromal damage through chemotherapy-induced ischemia and inflammation can indirectly affect ovarian endocrine function [24,29]. Ovarian vascular injury, as seen after treatment with vinca alkaloids, can be an indirect reason why the follicle count lowers. Vinca alkaloids inhibit tubulin polymerization and cause disruption of microtubules during mitosis [17,24]. This suppression of microtubule dynamics leads to vascular impairment and fibrosis, contributing to ovarian failure. Ovarian vascular injury is also described for other chemotherapeutics, such as alkylating agents [24]. 

##### Targeted Therapy

Over the past few decades, personalized medicine, based on the underlying molecular and genetic characteristics of the tumour, has made its way into cancer treatment. These targeted therapies have not only improved the overall survival of oncology patients but also generally have less toxicity compared with conventional chemotherapy [36]. The literature on potential fertility-related side effects is sparse, but this manuscript offers some insight into the potential gonadal effects of these treatments [37].

*Monoclonal antibodies*

Monoclonal antibodies against human epidermal growth factor receptor 2 (HER2; also known as ErbB2), e.g., trastuzumab and pertuzumab, are widely used in HER2-positive breast cancer and more recently also in other HER2-positive cancer types such as gastro-intestinal tumours [38,39,40]. The literature does not provide much information about the mechanisms of possible adverse ovarian side effects of monoclonal antibodies. The epidermal growth factor (EGF) signalling pathway plays an important role in the complex signalling network that regulates oocyte maturation and ovulation by transmitting the LH signal from the periphery to the follicle. HER2 (ErbB2) has no known ligand; however, it is a dimerisation partner for other ErbB receptors, including the EGF receptor (EGFR, also known as ErbB1). HER2 blockade could theoretically affect the rate of ovarian follicle activation and subsequent follicle growth [41].

*Tyrosine kinase inhibitors*

Tyrosine kinase inhibitors (TKIs) are a class of small molecule targeted therapy inhibiting cell signalling by blocking cell surface tyrosine kinase receptor phosphorylation. As many of these tyrosine kinases and receptors are involved in regulating oogenesis, primordial follicle activation, folliculogenesis and corpus luteum formation, inhibiting them may hamper normal ovarian function. The best-described tyrosine kinases and receptors that play a role in ovarian function are tyrosine-protein kinase (c-KIT), platelet-derived growth factor receptor (PDGFR), vascular endothelial growth factor (VEGF) and epidermal growth factor receptor (EGFR) [28,37].

Targeting the c-KIT receptor, expressed on the ovarian surface, leads to the inhibition of cell proliferation and apoptosis. PDGFR-a is found on primordial follicles, and TKIs that block the PDGFR-a receptor disrupt follicular development resulting in a decrease in total follicle count. These effects are reversible, and there are reports of women conceiving while on TKI, indicating that at least some gonadal function is preserved [42]. Although angiogenesis plays an important role in the development of gonads, preclinical studies found that TKIs with antiangiogenic activity (such as sorafenib, pazopanib or sunitinib) only have moderate effects on female fertility [13].

Another potential mechanism of indirect interference with normal ovarian function is the possible effect of TKIs on endocrine function, in particular, thyroid dysregulation, as this is associated with adverse fertility outcomes [28,43]. Inhibition of VEGF and EGFR may disrupt prolactin secretion and hypothalamic–pituitary–ovarian axis functioning [37]. 

Bussies et al. add, however, that kinase inhibitors that target mTOR (mammalian target of rapamycin complex 1) or the BCR-ABL (breakpoint cluster region-abelson) fusion protein seem to be protecting the ovarian reserve in patients undergoing chemotherapy. Inhibition of mTOR appears to temporarily halt ovarian cell activation making them less vulnerable to the toxic effect of chemotherapy [37].

The effects of TKIs on ovarian function seem to be reversible after treatment interruption, but an optimal wash-out period is unclear [42]. Interestingly, there are some hypotheses that the ovaries may be able to compensate for these inhibitory effects through the activation of alternative signalling pathways [37].

*Other*

Poly ADP-ribose polymerase (PARP) inhibitors are used in BRCA-mutated cancers and interfere with DNA damage repair as PARP enzymes repair single-strand DNA breaks. It is possible that PARP inhibition causes loss of primordial follicles due to loss of DNA repair and therefore impairs fertility [28].

Proteasome inhibitors, matrix metalloproteinases, heat shock protein inhibitors, and promotors of apoptosis are relatively new classes of small molecule inhibitors. The potential effects of these agents on the female reproductive system are not described [37].

##### Immunotherapy

The discovery of immunotherapy with immune checkpoint inhibitors (ICI) has improved the survival of oncology patients, both in metastatic and adjuvant settings [44]. ICIs are agents that block immune checkpoint proteins, such as programmed death-1 (PD1), programmed death ligand-1 (PDL1) and cytotoxic T-lymphocyte-associated protein 4 (CTLA4). Today, they are incorporated into the standard treatment of several malignancies, such as melanoma and triple-negative breast cancer [45,46]. The mechanism of action relies on releasing the brake on the immune system, which initiates and enhances the antitumor immune response [47]. ICIs in the adjuvant setting significantly reduce the risk of relapse, and in the metastatic setting, complete responses are described [48,49]. These favourable outcomes inevitably affect the patient’s perspective and desire for family planning. Despite the rapid expansion of indications for treatment with ICI, very little data are available to date on the effect of these drugs on fertility [50,51,52].

Inherent to treatment with ICI are immune-related adverse events (irAEs), which can affect basically any organ, including the endocrine system [53]. Endocrine immune-related adverse events, such as hypothyroidism or hypophysitis, are well-described, potentially leading to ovarian insufficiency by reducing gonadotropin secretion [13,22,28,31,37,54]. Next to the possible endocrine side effects, Alesi et al. describe the hypothesis that immunotherapy may have an impact on fertility by altering circulating immune cells, for example, macrophages, T-lymphocytes, mast cells and neutrophils and elevation of cytokines, such as tumour necrosis factor α and interleukin 1 [28]. However, the mechanisms behind this hypothesis are not fully understood. 

#### 3.1.3. Gonadotoxic Effect of Systemic Oncological Treatments

##### Chemotherapy

The following sections describe the gonadotoxic effects of each class of chemotherapy. Table 1 shows the different agents of chemotherapy and summarises their associated risk of female gonadotoxicity.

*Alkylating agents*

Alkylating agents (such as cyclophosphamide, ifosfamide, melphalan and busulfan) significantly contribute to POI and infertility and are considered to be the most gonadotoxic chemotherapeutics [61]. Their toxicity is dose-dependent, and even at very low doses, detrimental effects on ovarian function have been described [23]. There is also a relationship between dose, age and gonadotoxicity: a lower dose at an older age can cause gonadotoxicity at a more rapid pace than a slightly higher dose at a younger age [18]. In general, regimens containing alkylating agents resulted in lower AMH levels and less recovery of AMH post-treatment than in regimens without alkylating agents [16]. 

Van den Berg et al. report that the risk of fertility impairment is related to the cyclophosphamide equivalent dose (CED) score, expressing the total alkylating agent exposure. This score reflects the clear dose-effect relationship with alkylating-containing regimens and survivors with a CED score below 7121 mg/m^2^ did not seem to be at significantly increased risk of fertility impairment. Results of this analysis in female childhood and AYA cancer survivors showed that cumulative doses of the following treatments were significantly associated with fertility impairment: busulfan (any dose), carmustine, cyclophosphamide, melphalan (>140 mg/m^2^) and procarbazine (>5600 mg/m^2^) [62].

In early breast cancer, cyclophosphamide is one of the cornerstone chemotherapeutics [63]. Patients with cyclophosphamide-containing regimens have a significantly higher risk of POI compared with those without, and the chance of amenorrhea is twice as high [64]. The highest risk of amenorrhea (>80%) is described for CMF, CEF, CAF and TAC for six cycles in women older than 40 years. The same regimens in women between 30 and 39 years old have an intermediate risk of amenorrhea (40–60%) and a low risk (<20%) in women younger than 30 years. Patients treated with four cycles of AC or EC have an intermediate risk for amenorrhea (40–60%) when aged above 40 years old and a low risk (<20%) when they are younger than 40 years [60]. A large analysis of breast cancer patients treated with alkylating agents (specifically with the regimen CMF), including more than 2500 women, reported a risk of treatment-related amenorrhea (TRA) of 40% for women ≤40 years of age and 76% for women ≥41 years of age [65]. Another study in premenopausal breast cancer patients treated with CMF reported amenorrhea in 61% of women younger than 40 years and 95% of women older than 40 years [66]. The median onset time of TRA has been reported to be 6–16 months in women younger than 40 years and 2–4 months in women older than 40 years [22]. So, in older women, amenorrhea occurs at a shorter interval after chemotherapy initiation. In addition, amenorrhea is more likely to be irreversible [14]. Other cyclophosphamide-containing regimens for breast cancer, such as CEF and CAF, are reportedly even more gonadotoxic, with rates of amenorrhea at 1 year of 40–50% in women younger than 40 years old and rates of 90–100% in women older than 40 years old [67]. In breast cancer patients, the trend is to modify the chemotherapy regimens by reducing cyclophosphamide as much as possible in order to result in less gonadotoxicity [23]. The NSABP B-30 trial compared combinations of doxorubicin 50 mg/m^2^ (A), docetaxel 75 mg/m^2^ (T) and cyclophosphamide 500 mg/m^2^ (C). They reported amenorrhea rates at 12 months of 37.9% for AT, 57.7% for TAC and 69.8% for AC, followed by T [68]. The more modern anthracycline-based regimens usually combine a lower dose of cyclophosphamide with anthracyclines, reducing the risk of POI [31]. Dose-dense schedules do not seem to increase the risk of TRA [25]. 

In female non-Hodgkin’s lymphoma patients treated with five to six cycles of CHOP, 63% had the return of menstrual cycles 3 months after completing treatment [69]. Others report >90% menses recovery for patients between 30–45 years treated with rituximab and CHOP [32]. To conclude, the risk of permanent ovarian failure is low when using less than six cycles of CHOP [58]. BEACOPP, the escalated regimen for Hodgkin’s lymphoma patients, is associated with irreversible infertility [54]. Menses recovery was seen in 82% of female patients younger than 30 years old treated with six to eight cycles of BEACOPP, but only in 45% of women between 30 and 45 years old [70]. A longitudinal study including 66 female patients reported significant decreases in AMH levels for patients treated with BEACOPP, but there was no relation with the number of cycles [55].

*Anthracyclines*

Anthracyclines are classified as chemotherapeutic agents with low to intermediate gonadotoxic risk and are widely used in breast cancer, lymphoma and sarcoma treatment [63,71]. 

Modern breast cancer chemotherapy regimens include anthracyclines. However, their role continues to evolve, mainly due to the risk of cardiotoxicity and secondary leukaemias [31]. The effects of anthracyclines alone on fertility are unknown, but there is a low to intermediate risk of TRA for anthracycline-containing regimens, such as AC or EC, due to lower cumulative doses of cyclophosphamide [31]. The CALGB trial, in which premenopausal breast cancer patients were treated with six cycles of CAF with different doses of doxorubicin, reported an amenorrhea rate of 51%, but they did not observe an association with dose intensity [72]. In parallel, Venturini et al. reported amenorrhea rates of 64% in breast cancer patients treated with CEF, independently of dose-dense scheduling [73]. In addition, Lambertini et al. did not observe an increased risk of TRA with dose-dense chemotherapy, but they did report a significantly improved overall survival [74]. These studies support the hypothesis that dose-dense scheduling does not increase the risk of amenorrhea. Another study using three to six cycles of CEF with increasing doses of epirubicin (50 mg/m^2^, 75 mg/m^2^ or 100 mg/m^2^) did report a relationship between cumulative dose (less than 300 mg/m^2^, 300–450 mg/m^2^ and greater than 450 mg/m^2^) and amenorrhea rates. They reported that in these three treatment groups, 52%, 58%, and 69% of women experienced amenorrhea [22]. 

Early-stage Hodgkin’s lymphoma can be treated with ABVD (doxorubicin, bleomycin, vinblastine and dacarbazine) [71]. A small study in women treated with ABVD and radiotherapy showed transient amenorrhea in 33% of women under 45 years of age. In women younger than 25 years old, there was no ovarian failure [75]. Behringer et al. report menses recovery in more than 90% of women between 30 and 45 years old after ABVD induction [70]. Recovery of AMH level after treatment with ABVD was complete after 1 year, according to Decanter and colleagues [76]. In general, ABVD treatment is not associated with a greater risk of premature menopause and, as shown in these studies, is considered a low-risk gonadotoxic treatment [13,58].

*Taxanes*

The addition of taxanes in treatment regimens for breast cancer allowed the lower total dose of anthracyclines because of the introduction of sequential regimens [63].

Martin et al. compared TAC with FAC as an adjuvant treatment for early node-positive breast cancer and demonstrated a superior overall survival for TAC. Unfortunately, TAC was associated with a higher rate of amenorrhea compared with FAC (61.7% versus 52.4%) [77]. In addition, Silva et al. [78] described a lower rate of menses recovery in women who received taxanes compared with those not exposed to this agent, and Lambertini et al. [79] found that the addition of taxanes to anthracycline-based chemotherapy increases the risk of TRA. Another study by the same research group observed no adverse reproductive health outcomes in two patients treated with weekly paclitaxel [30]. 

In more modern regimens, taxanes are given sequentially rather than concurrently with AC [63]. A small retrospective study of 159 premenopausal patients receiving adjuvant anthracycline-based chemotherapy did not observe a higher rate of amenorrhea in those treated with a sequential taxane compared with those not receiving a taxane [80]. In contrast, another retrospective study described a higher risk of permanent amenorrhea for patients treated with AC followed by T compared with AC alone. This risk was comparatively more pronounced in women above 40 years old [81]. To conclude, the gonadotoxic risk of taxanes is not well defined, and the results are conflicting.

*Platinum agents*

Platinum drugs appear to have a moderate risk of damaging the ovarian reserve [23]. A small retrospective study comparing AC-T with TC (docetaxel/carboplatin) in breast cancer patients reported a 13% risk for TRA in patients treated with TC compared with 51% in AC-T treatment [82]. Lambertini et al. reported a higher rate of TRA in HER2-positive patients treated with TCH (docetaxel/carboplatin/trastuzumab) compared with anthracycline-based therapy [79]. However, none of these studies determines the individual risk of platinum agents.

*Antimetabolites*

The use of methotrexate and 5-fluorouracil in breast cancer treatment regimens has not been associated with higher rates of amenorrhea [27]. Santaballa et al. describe no severe effects on fertility for treatment with 5-fluorouracil or capecitabine [13].

##### Targeted Therapy

*Monoclonal antibodies*

For trastuzumab, the risk of gonadotoxicity is considered to be low based on the following data. First, Morarji et al. [83] report a significantly higher serum AMH level in patients treated with chemotherapy and trastuzumab compared with chemotherapy alone. Second, a retrospective analysis of 431 premenopausal patients treated with anthracycline- and taxane-based chemotherapy +/− trastuzumab reported that the addition of trastuzumab did not alter the chance of menses recovery [22]. Third, a retrospective analysis of premenopausal breast cancer patients in the APT trial (Adjuvant Paclitaxel Trastuzumab trial) showed rates of amenorrhea in 28% of patients at 1 year. How much of this was driven by paclitaxel versus trastuzumab is unknown [84]. Fourth, an analysis of the ALTTO trial (Adjuvant Lapatinib and/or Trastuzumab Treatment Optimisation study, a phase III clinical trial of two targeted therapies for HER2-positive breast cancer) reported an incidence of amenorrhea of 72.1% for trastuzumab monotherapy, 72.1% for trastuzumab followed by lapatinib (oral anti-HER2 therapy), 74.8% in the trastuzumab plus lapatinib and 74.0% for the lapatinib monotherapy. There was no control arm without anti-HER2 therapy, and the rates of amenorrhea will be influenced by previous chemotherapy exposure. However, in the combination group, the amenorrhea rates do not differ from the monotherapy groups suggesting the safety of HER2-directed agents [79]. Last, Lambertini et al. recently published data from the NeoALTTO trial where patients with HER2-positive early breast cancer were treated with neo-adjuvant anti-HER2 therapy alone (trastuzumab and/or lapatinib) for 2 cycles (6 weeks total) and then together with weekly paclitaxel for 12 cycles. So, this study was able to identify the acute gonadal impact of anti-HER2 therapy without the influence of prior anthracycline/cyclophosphamide-based chemotherapy. They observed a small reduction in AMH levels during 2 weeks of anti-HER2 treatment alone and then a profound decline to almost undetectable levels in most patients after the addition of weekly paclitaxel. There was no difference between trastuzumab and lapatinib neither in the group with a double-HER2 blockade. This study does not provide information about the long-term effects on ovarian reserve [85]. There are no studies that assessed the impact of pertuzumab (HER2–HER3 blockade) or neratinib (an oral anti-HER2 drug) on fertility [22,25,31].

The ATEMPT trial compared adjuvant treatment in HER2-positive breast cancer with paclitaxel plus trastuzumab versus TDM-1 (ado-trastuzumab emtansine, an antibody–drug conjugant). In the TDM-1 group, 75% of premenopausal women had menses at 18 months compared with less than 50% in the control arm, suggesting a potential gonadal safety for TDM-1 [86].

About bevacizumab, a monoclonal antibody against VEGF used in cervical cancer and colon cancer [22], Lorenzi et al. mention transient amenorrhea (more than 3 months) and increased FSH. The long-term effects are unknown [87]. A trial by Allegra et al. reported an incidence of ovarian failure (defined as amenorrhea for ≥3 months with blood FSH levels ≥30 mIU/mL) of 34% when bevacizumab was administered and an incidence of 2% in the control group. Only 22% of women recovered ovarian function after treatment cessation [88].

Rituximab, a monoclonal antibody targeting the CD20 antigen, is widely used in haematological malignancies. The addition of rituximab to chemotherapy did not increase the risk of impaired ovarian function, especially in women younger than 40 years old [89].

*Tyrosine kinase inhibitors*

Regarding the fertility effects of imatinib (BCR-ABL tyrosine kinase inhibitor), results are scarce for women. There is only one case report of POI in chronic myeloid leukaemia (CML) patients receiving high-dose imatinib [90]. Bussies et al. found that imatinib induces oligomenorrhea [37]. Another report by Lorenzi et al. describes the lower ovarian response to GnRH stimulation for fertility preservation and also mentions increased FSH levels and lower AFC [87]. To conclude, the risk of imatinib is still uncertain. Data on other BCR-ABL tyrosine kinase inhibitors, nilotinib and dasatinib, suggest that they do not affect gonadal function [13]. Others conclude that there is not enough information about the effect of dasatinib on fertility [42].

Pazopanib (TKI with anti-angiogenic activity) was also found to induce oligomenorrhoea; however, discontinuation of treatment led to a return of regular menses within 2 months [91]. A clinical study for gefitinib (EGFR TKI) shows suppression of androgen levels in men and women, but there are no further data on the effect on fertility [13]. No clinical data are reported in women about crizotinib (ALK-receptor TKI), sorafenib (TKI with anti-angiogenic activity) and erlotinib (EGFR TKI) [13].

*Others*

Regarding BRAF-MEK inhibitors, protein kinase inhibitors used in BRAF-mutated melanoma, such as dabrafenib and trametinib, their impact on the fertility of women is unknown [22].

Olaparib, a PARP inhibitor, does not appear to interfere with fertility [22]. In addition, it does not seem to aggravate gonadotoxicity induced by chemotherapy [25].

The fertility effects of cyclin-dependent kinase (CDK) 4/6 inhibitors remain unknown. These are protein kinase inhibitors involved in cell cycle regulation [25,28].

##### Immunotherapy

There is no evidence for primary hypogonadism in women caused by immunotherapy [50]. In comparison, there are reports of hypophysitis that can cause secondary hypogonadism. These endocrine side effects are chronic and require hormonal replacement therapy. The rate of hypophysitis is 5.6% for ipilimumab (CTLA4-inhibitor), 0.5% for nivolumab (PD1-inhibitor), 1.1% for pembrolizumab (PD1-inhibitor) and 8.8–10% for the combination [92]. The frequency of potential isolated hypogonadotropic hypogonadism is unknown. Underestimation of immunotherapy-related hypogonadism is plausible as sex hormones are not routinely tested [50]. In addition, these endocrinological irAEs are more frequently reported in premenopausal women, with thus a higher risk of fertility problems secondary to treatment with ICI [93]. So far, there are no clinical studies evaluating the fertility risks of immunotherapy [28,50,54].

### 3.2. Men

#### 3.2.1. Spermatogenesis and Measuring Gonadal Function

Spermatogenesis takes place in the seminiferous tubules of the testes, and the required time to develop mature spermatozoa is 74 days. This process begins in puberty and continues throughout adult life. The male primordial germ cells or spermatogonia are located in the seminiferous tubules and are mitotic cells up until they differentiate to initiate meiosis. When meiosis takes place, haploid spermatids are produced, and these differentiate further into spermatozoa. Subsequently, sperm maturation is completed in the epididymis. Sertoli cells, located in the seminiferous tubules supporting the germ cells, play an important role in promoting spermatogenesis in response to testosterone, secreted by Leydig cells and FSH [13].

Semen analysis is the cornerstone of male fertility assessment by evaluating sperm volume, sperm concentration, motility and morphology of the spermatozoids. The impact of systemic oncological therapies on spermatogenesis can vary between azoospermia (absence of sperm cells), oligozoospermia (decreased sperm concentration), asthenozoospermia (reduced sperm motility) or teratozoospermia (increased abnormal forms of sperm) [13]. When semen analysis is not available, gonadotropin (FSH and LH) levels, testosterone levels, and inhibin B can contribute to an indirect evaluation of fertility. The loss of germ cells leads to decreased secretion of inhibin B by the Sertoli cells and subsequently increases FSH levels, which are secreted by the pituitary gland. Inhibin B and FSH are thus part of a negative feedback loop on the hypothalamic–pituitary–gonadal axis [94].

In general, gonadotoxicity is expressed as the risk of developing prolonged azoospermia. It is divided into three classes: high risk, intermediate risk and low to very low risk, with a chance of prolonged azoospermia of more than 75–80%, 25–75% and less than 25%, respectively [13,58].

#### 3.2.2. Mechanisms of Gonadotoxicity

##### Chemotherapy

**Figure 3:**
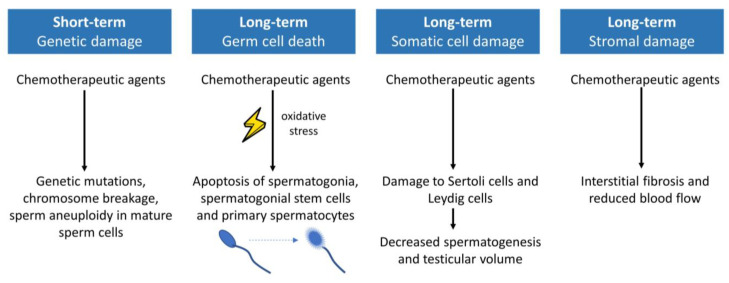
Mechanisms of gonadotoxicity of chemotherapy in men.

Chemotherapy can cross the blood–testis barrier and is known to cause short-term and long-term harmful effects on male fertility [95]. About 25% of men with cancer diagnosis and treatment report fertility problems, ranging from mild oligozoospermia to persistent azoospermia [96,97]. The degree of gonadotoxicity depends on the primary semen parameters, the drug administration route and the type and cumulative dose of the administered chemotherapeutics. Infertility after chemotherapy exposure (Figure 3) can be the result of direct injury to germ cells or indirect damage to endocrine and paracrine control of somatic cells [98].

First, infertility can be caused by direct injury to germ cells, depending on the phase of spermatogenesis at the time of drug administration [99]. The proliferating type B spermatogonia are easily targeted by chemotherapy agents. In contrast, type A spermatogonia (spermatogonial stem cells) have only minimal mitotic activity and are, therefore, more resilient to chemotherapeutic agents [95]. Some sperm cells will be able to repair DNA damage and survive [99]. Similar to women, alkylating agents have the most detrimental effects on spermatogenesis. Cyclophosphamide and cisplatin cause DNA damage leading to cell death of spermatogonia, primary spermatocytes and spermatogonial stem cells. Apoptosis is the primary mechanism of cell death for cyclophosphamide, cisplatin, etoposide and vincristine. Doxorubicin also causes testicular damage by induction of necrosis or autophagy [99]. Next to sperm DNA damage or fragmentation, chemotherapy can cause genetic mutations, chromosome breakage and sperm aneuploidy [13]. These DNA changes can be long-lasting after chemotherapy, with some studies reporting abnormalities even 2 years after treatment, suggesting that the effects of cancer drugs on spermatogonial stem cells have a long-term impact [98].

Mature sperm cells are more resistant to chemotherapeutic depletion but highly sensitive to DNA damage because spermatids lack DNA repair mechanisms [13,95]. The greatest risk of genetic damage by alkylating agents starts on day one of treatment and lasts for at least one spermatogenic cycle (75 days) [13]. So, sperm DNA integrity can be affected after a single treatment [8]. For topoisomerase II inhibitors, the greatest damage occurs between 30 and 50 days after administration, as they act mainly on meiosis [13]. A time frame of 7–10 weeks has been described for nucleoside analogues, antimetabolites and bleomycin [99].

Second, impairment of somatic cells and endocrine or paracrine testicular functions can contribute to infertility. Data on the impact of chemotherapy on somatic cells in the testes are limited to Sertoli cells and Leydig cells. Macrophages also play a role in fertility, but their role in gonadotoxicity caused by chemotherapy is not investigated [98]. Sertoli cell dysfunction leads to decreased spermatogenesis and is associated with elevated FSH levels and a decrease in testicular volume due to germ cell depletion [13]. Chemotherapy-induced damage to Leydig cells can temporarily or permanently decrease testosterone production and is associated with elevated LH levels [13].

Next to Sertoli cell and Leydig cell dysfunction, interstitial fibrosis and reduced blood flow in the testis are described as a consequence of chemotherapy [98,99]. Another cause of testicular dysfunction is the oxidative and endoplasmic reticulum (ER) stress triggering testicular cell apoptosis [99]. Additionally, sperm mobility can be disturbed, as shown by an in vitro study performed on human sperm where doxorubicin and vincristine had a sperm-immobilizing activity [98].

Semen volume, sperm concentration, sperm motility and sperm viability are semen parameters that are affected by chemotherapeutic agents [99,100]. Changes in the histological structure of the seminiferous tubule and decreased testicular weight are also described. Some of those effects are reversible, mostly depending on the cumulative chemotherapy dose [98,99].

##### Targeted Therapy

As parallel for women, the c-KIT and PDGFR pathways are important for testis development, hormone production and spermatogenesis. Inhibition of these pathways by TKIs can result in decreased spermatogenesis. PDGFR is expressed by Leydig cells, and blocking the PDGFR pathway by treatment with imatinib can induce apoptosis of these cells, leading to lower testosterone concentrations [42]. Most case reports have shown that the overall effects of TKIs on male fertility are reversible. Second-generation TKIs may not cross the blood–testis barrier and therefore have minimal effects on male fertility or testosterone production [42]. Angiogenesis is crucial for gonadal development, but the influence of anti-angiogenic agents is unclear. Preclinical studies report only moderate gonadotoxic effects of TKIs with antiangiogenic activity (sunitinib, sorafenib or pazopanib) [13].

##### Immunotherapy

As for women, the risk of gonadotoxicity of ICIs in men remains unclear. IrAEs, such as hypothyroidism or hypophysitis, can cause decreased testosterone levels due to secondary hypogonadism [13]. Unlike women, some data suggest that ICIs can also cause primary hypogonadism in men [50].

#### 3.2.3. Gonadotoxic Effect of Systemic Oncological Treatments

##### Chemotherapy

The following sections describe the gonadotoxic effects of each class of chemotherapy. Table 2 shows the different agents of chemotherapy and summarises their associated risk of male gonadotoxicity.

*Alkylating agents*

Alkylating agents have the most detrimental effects on fertility in a dose-dependent manner and are used in AYAs, for example, in sarcoma and lymphoma patients. Cyclophosphamide reduces sperm parameters (viability, motility and count), decreases testicular weight, and increases sperm abnormalities. In addition, it is associated with increased FSH levels, reduced inhibin B levels and reduced testosterone levels [99].

Sarcoma patients treated with a combination of cyclophosphamide, doxorubicin and dacarbazine with or without vincristine developed azoospermia within 4 months of therapy. Five years after finishing chemotherapy, 40% of patients had normal sperm parameters. The cumulative dose of cyclophosphamide was the major risk factor for gonadotoxicity, with only 10% recovering to normospermic levels when doses exceeded 7.5 g/m^2^ [101].

In non-Hodgkin’s lymphoma (NHL) patients treated with CHOP, 22% of men were found to be azoospermic 2 years after treatment [58]. Pallotti et al. reported that 90% of patients with first-line treatment for NHL will recover spermatogenesis. Most patients received R-CHOP in the first line [95]. Treatment with cyclophosphamide, doxorubicin, vincristine, prednisone and bleomycin (ACVBP) induces azoospermia in all treated patients. Seven years after treatment, two-thirds of patients regained normospermia [102].

In male Hodgkin’s lymphoma patients treated with BEACOPP, 89% will become azoospermic with higher levels of permanent azoospermia in patients who received 6–8 cycles compared with 2–4 cycles. When 2–4 cycles were used, spermatogenesis could return 3–4 years post-treatment [103]. BEACOPP is more gonadotoxic than ABVD, given the inclusion of alkylating agents in this scheme. Van der Kaaij et al. reported an FSH level recovery time of 18 months for recovery in 82% of men treated without alkylating agents, compared with 27 months for recovery in 30% of men treated with alkylating agents [104].

*Platinum agents*

Cisplatin is known to decrease sperm parameters and induce alterations of FSH, LH and testosterone. On top, it is associated with a decrease in testicular volume [99].

Platinum agents are the cornerstone of testicular cancer treatment. A study in patients with testicular cancer treated with cisplatin and/or carboplatin described an increase in sperm DNA alterations and aneuploidy at 12 and 24 months after treatment [99]. Santaballa et al. reported a declined pregnancy rate in female partners of male patients treated with bleomycin, etoposide and cisplatinum (BEP) [13]. In fact, one year after treatment, patients who have been treated with more than two BEP cycles displayed a lower total sperm count, whereas men treated with one or two BEP cycles had a normal sperm recovery after testicular cancer treatment [105]. Lampe et al. describe a study where 80% of patients treated with cisplatin for testicular cancer recovered spermatogenesis 5 years after treatment. The number of cycles was also found to be a risk factor for the non-recovery of normal sperm parameters [106].

*Anthracyclines*

Anthracyclines cause abnormalities in hormone levels and decrease sperm parameters [99]. In male Hodgkin’s lymphoma patients treated with ABVD, 40% had azoospermia at 6 months after therapy. At 6 and 12 months, FSH was raised compared with healthy controls. Two years after treatment, 57% of patients regained normal sperm parameters [103]. Others reported normospermia after 18 months [107].

*Taxanes*

As for women, data on the gonadotoxic effects of taxanes are limited. A study of male patients who received taxane-based chemotherapy showed reduced inhibin B levels and elevated FSH levels in all patients during chemotherapy. A significant number of patients had a reduction of testicular volume. No data are available on recovery after treatment [94].

##### Targeted Therapy

Some data are available on the fertility effects of imatinib. This BCR-ABL tyrosine kinase inhibitor reduces the total number of spermatogonia, but it does not affect spermatogenesis, suggesting that future fertility is preserved. For example, a study in CML patients treated with imatinib showed reduced sperm density, sperm counts, survival rates and activity, and it confirmed that imatinib crosses the blood–testis barrier. However, imatinib did not affect the structure of the testes or the sex hormone levels [108]. In addition, many case reports describe pregnancies in female partners of men treated with imatinib. Next to the impaired sperm counts, others describe a decrease in Leydig cell counts in 90% of patients and a reduction in free testosterone concentration in 73% of patients. This reduction in testosterone seems reversible [42]. For male partners looking to conceive, a washout period of 72 days is proposed to allow for sperm maturation [109]. Unlike in adults, there are case reports describing severe oligospermia in males treated with imatinib prior to puberty, suggesting an irreversible effect on fertility [42]. Nilotinib and dasatinib, second-generation BCR-ABL TKIs, do not pass the blood–testis barrier, and, therefore, they have minimal effects on fertility [42].

A clinical study for gefitinib (EGFR TKI) shows suppression of androgen levels in men and women, but there are no further data on the effect on fertility [13]. Clinical data on crizotinib are limited. One study found lower testosterone levels in men, and Loren et al. describe an association with hypogonadism [13,35]. The clinical effects of bosutinib (BCR-ABL TKI), erlotinib (EGFR TKI), rituximab, brentuximab-vedotin and TKIs with antiangiogenic activity (such as sunitinib, sorafenib and pazopanib) are unknown [13,42,54].

##### Immunotherapy

In contrast to women, there are some case reports and case series describing orchitis with primary hypogonadism in men treated with ICI [110,111]. A small retrospective analysis of testicular biopsies of patients treated with anti-PD1 and anti-CTLA4 showed impaired spermatogenesis in six out of seven (85%) patients with melanoma [112]. Another study analyzed sperm specimens from 22 patients treated with ICI and found normal results in 82% of patients [113]. Peters et al. found that treatment with anti-PD1 and/or anti-CTLA4 was associated with low testosterone levels in 69% of the 49 men studied [114]. A case report describes a normozoospermic man who developed azoospermia 2 years after treatment with anti-PD1 and anti-CTLA4 [115]. To conclude, ICIs might cause primary hypogonadism, but the incidence, extent and duration are unclear.

As for women, it is known that ICIs can cause hypophysitis, with the highest incidences for the combination of anti-PD1 and anti-CTLA4 [50]. One case of isolated hypogonadotropic hypogonadism has been described [116]. These endocrine side effects are mostly permanent. The frequency of isolated hypogonadotropic hypogonadism is unknown, and the diagnosis can be difficult because sex hormones are lacking in routine testing. In addition, the effect on fertility of these pituitary side effects remains uncertain [50]. At last, one study demonstrated that men treated with anti-PD1 showed a significant decrease in FSH levels from baseline to week 12. No significant differences were observed in LH or testosterone levels [117].

## 4. Discussion

This review aims to provide oncologists, reproductive endocrinologists and gynaecologists with a practical overview of the current knowledge about the impact of systemic oncological treatments on the fertility of female and male adolescents and young adults. For chemotherapy, the effects on fertility are well described, and it is known that alkylating agents have the most detrimental consequences for female and male fertility. Additionally, other classes of chemotherapy, such as anthracyclines and platinum agents, have an intermediate risk of prolonged amenorrhea in women and permanent azoospermia in men. In general, chemotherapy-induced gonadotoxicity is dose-dependent. For women, the risk of gonadotoxicity is also depending on gonadal reserve before treatment and patient age. Regarding the fertility effects of targeted therapy and immunotherapy, data are too limited to draw conclusions. The potential mechanisms are still under investigation, and there is an important lack of clinical data. Additionally, the effects of each TKI or ICI can be different, so results cannot be extrapolated, and every new treatment needs to be investigated on an individual basis.

Measuring the gonadal effect of oncologic treatments is difficult as several tests are available, but none have shown to be the absolute standard for measuring gonadotoxicity. In light of this, studies are heterogenic because of the lack of well-standardized outcomes and definitions of infertility. Some studies report hormonal outcomes, some report ultrasound measurements, and others report clinical outcomes such as loss of menses or azoospermia, which are surrogate measures of infertility. For women with treatment-related amenorrhea, it is important to know that even after the resumption of menses, they can still be at risk for POI. Therefore, it is important to distinguish between short- and long-term results, but studies often lack a prolonged follow-up.

Healthcare practitioners may experience difficulties estimating the fertility risk for their cancer patients because of the heterogeneity of study results, patient-specific factors and constantly evolving and heterogeneous treatment regimens. Despite the difficulties in assessing the risk, it is extremely important to adequately inform a patient about the risk of infertility and possible long-term health problems, such as POI. Husson et al. described the wide range of 17% to 83% of AYA patients with haematological malignancies that had a discussion about the potential impact of their treatment on fertility and the possibilities of fertility preservation before initiating treatment. Qualitative research also showed that AYAs reported unrealistic expectations and uncertainties regarding their reproductive potential after treatment which negatively impacted their quality of life [6]. These results show that healthcare practitioners should be more educated about reproductive health, the impact of oncological treatments on fertility, POI and the possible fertility preservation strategies to adequately inform and refer their patients to an oncofertility consultation program. Current options for female fertility preservation include oocyte, embryo or ovarian tissue cryopreservation [7,8,118]. For male patients, the standard of care is sperm cryopreservation [8,118].

Next to assessing and investigating the possible risk of oncological treatments, researchers are looking for possible preventive strategies for reducing chemotherapy-induced gonadotoxicity. One of the most controversial topics is the use of gonadotropin-releasing hormone analogues (GnRHa) for preventing POI. The hypothesis states that downregulation of FSH production can prevent the accelerated primordial follicle recruitment and thus prevent the second hit to the ovaries and preserve the ovarian reserve. Side effects of GnRHa are related to low estradiol levels [31,119]. Data are contradictory with the benefit for ovarian reserve in women with breast cancer, but no effect was shown in lymphoma patients [35]. ESHRE (European Society of Human Reproduction and Embryology) guidelines mention that GnRHa can be considered in premenopausal breast cancer patients for ovarian function protection, but this strategy should not be considered to replace other fertility preservation techniques [7].

Other fertoprotective agents under investigation in women are TKIs, such as imatinib, mTOR inhibitors, G-CSF, anti-oxidants, tamoxifen and Sphingosine-1-phosphate [120]. Some protectants (e.g., multidrug resistance gene 1) are designed to reduce drug delivery to the ovary. Others (e.g., TKI and mTOR inhibitors) protect against ovarian follicle death or vascular injury (e.g., G-CSF). Currently, there is not much focus on preventing stromal damage, although the stromal’s functioning is vital to follicle health. Of course, it is important that these agents will not interfere with anti-cancer treatments. On imatinib, data on the efficacy against cisplatin-induced damage have been conflicting, with some studies finding protective effects and others finding either no evidence of protection or even harmful effects [120,121]. A possible explanation for these contradictory results could be that imatinib can target multiple tyrosine kinase signalling pathways that play a regulatory role in the ovary. Only a few of these fertoprotectants are studied in human ovarian tissue, including tamoxifen and Sphingosine-1-phosphate, both aiming to prevent the loss of primordial follicles [120]. There may also be a role for DNA repair agents to enhance DNA repair in primordial follicle oocytes, making them more resistant to chemotherapy-induced death and leading to fertility preservation and prevention of ovarian ageing [29]. BRCA 1 and 2 are tumour-suppressor genes playing an essential role in homologous DNA recombination and double-strand DNA break repair. Studies showed that women with BRCA mutations, whose oocytes are deficient in double-strand DNA break repair, exhibit accelerated ovarian ageing and are more likely to suffer from POI after chemotherapy [29,122]. Such protectants are of huge interest not only to preserve fertility but also to enable women to maintain endocrine function and avoid the adverse health effects of POI.

## 5. Conclusions

Fertility preservation in AYAs with cancer is one of the major issues when dealing with the long-term sequellae of cancer treatment. Timely referrals to oncofertility services to maximize the quality of life of young cancer patients are of utmost importance, and it is essential for physicians to be well-informed about the impact of oncological treatments on gonadal function and fertility, as well as the different fertility preservation techniques. This review provides physicians with a practical overview of the current knowledge about the impact of systemic oncological treatments on the fertility of female and male AYAs, including targeted therapy and immunotherapy. While the effects of chemotherapy on fertility are meanwhile fairly understood, insufficient data are available on the fertility effects of targeted therapy and immunotherapy.

This work also underlines the need to further explore the possible harmful effects of systemic oncological treatments on fertility. This unmet need will become increasingly important given the continuously evolving treatment regimens, for example, the introduction of ICIs in the (neo-)adjuvant oncological setting. In this regard, it would be useful to include uniform fertility outcomes as subanalyses in future clinical trials investigating new and existing treatments to determine the long-term fertility effects. Moreover, it would be of great value to track the application and outcomes of fertility preservation strategies in (inter)national registries. Additionally, data relating to AYA patients remain scarce as they are often underrepresented in clinical trials. Not only to make progress in the treatment and survival of their oncological disease but also regarding the topic of fertility, special attention needs to be given to the inclusion of AYAs in future research.

## Figures and Tables

**Table 1:** Gonadotoxic risk associated with chemotherapy agents in women [13,14,15,17,18,22,23,24,25,27,54,55,56,57,58,59,60]. This table contains examples and is not a complete list.

	High Risk (>80%)	Intermediate Risk (20–80%)	Low/Very Low Risk (<20%)	Unknown Risk
Alkylating Agents				
Cyclophosphamide	✓			
Carmustine	✓			
Ifosfamide	✓			
Busulfan	✓			
Chlorambucil	✓			
Melphalan	✓			
Procarbazine	✓			
Nitrogen mustard	✓			
Antimetabolites				
Cytarabine		✓	✓	
Methotrexate			✓	
Mercaptopurine			✓	
Fluorouracil			✓	
Gemcitabine			✓	
Antimitotic Cytostatics				
Vinblastine		✓	✓	
Vincristine			✓	
Taxanes		✓		✓
Anti-tumor Antibiotics				
Bleomycin			✓	✓
Dactinomycin			✓	
Daunorubicin			✓	
Doxorubicin		✓	✓	
Epirubicin			✓	
Mitomycin			✓	
Topo-isomerase Inhibitors				
Etoposide		✓		
Irinotecan				✓
Platinum-based Drugs				
Cisplatin	✓ >600 mg/m^2^	✓ <600 mg/m^2^		
Carboplatin		✓	✓	
Oxaliplatin		✓		✓
Combinations				
CMF (6 cycles) *	✓ >40 years old	✓ 30–39 years old	✓ <30 years old	
CEF (6 cycles) *	✓ >40 years old	✓ 30–39 years old	✓ <30 years old	
CAF (6 cycles) *	✓ >40 years old	✓ 30–39 years old	✓ <30 years old	
AC (4 cycles) *		✓ >40 years old	✓ <40 years old	
EC (4 cycles) *		✓ >40 years old	✓ <40 years old	
ABVD *			✓	
CHOP (4–6 cycles) *			✓	
TAC *		✓		
BEACOPP *		✓		
FOLFOX *		✓		
Anthracycline/ cytarabine			✓	
EURAMOS *	✓	✓		
EuroEWING 12 *	✓			

* Abbreviations: CMF, cyclophosphamide, methotrexate and fluorouracil. CEF, cyclophosphamide, epirubicin and fluorouracil. CAF, cyclophosphamide, doxorubicin and fluorouracil. AC, doxorubicin and cyclophosphamide. EC, epirubicin and cyclophosphamide. ABVD, doxorubicin, bleomycin, vinblastine and dacarbazine. CHOP, cyclophosphamide, doxorubicin, vincristine and prednisone. FEC, 5-fluorouracil, epirubicin and cyclophosphamide. TAC, docetaxel, doxorubicin and cyclophosphamide. BEACOPP, bleomycin, etoposide, doxorubicin, cyclophosphamide, vincristine, procarbazine and prednisolone. FOLFOX, fluorouracil, leucovorin and oxaliplatin. Euramos is a protocol for osteosarcoma consisting of methotrexate, cisplatin and doxorubicin. EuroEWING12 is a protocol for Ewing sarcoma consisting of different combinations of vincristine, ifosfamide, doxorubicin, etoposide, actinomycin D and cyclophosphamide.

**Table 2:** Gonadotoxic risk associated with chemotherapy agents in men [13,59,98]. This table contains examples and is not a complete list.

	High Risk (>75%)	Intermediate Risk (25–75%)	Low/Very Low Risk (<25%)	Unknown Risk
Alkylating Agents				
Cyclophosphamide (19 g/m^2^)	✓			
Carmustin (300 mg/m^2^)		✓		
Ifosfamide (42 g/m^2^)		✓		
Busulfan (600 mg/kg)		✓		
Chlorambucil (1.4 g/m^2^)	✓			
Melphalan (140 mg/m^2^)	✓			
Procarbazine (4 g/m^2^)	✓			
Antimetabolites				
Cytarabine (1 g/m^2^)			✓	
Methotrexate			✓	
Fluorouracil			✓	
Antimitotic Cytostatics				
Vinblastine (50 g/m^2^)			✓	
Vincristine (8 g/m^2^)			✓	
Anti-tumor Antibiotics				
Bleomycin			✓	
Dactinomycin		✓		
Daunorubicin			✓	
Doxorubicin (770 mg/m^2^)			✓	
Topo-isomerase Inhibitors				
Etoposide			✓	
Platinum-based Drugs				
Cisplatin	✓ >600 mg/m^2^	✓ <600 mg/m^2^		
Carboplatin (2 g/m^2^)			✓	
Combinations				
ABVD *			✓	
CHOP *			✓	
FOLFOX *				✓
BEACOPP *	✓			
EURAMOS *	✓			
EuroEWING 12 *	✓			

* Abbreviations: ABVD, doxorubicin, bleomycin, vinblastine and dacarbazine. CHOP, cyclophosphamide, doxorubicin, vincristine and prednisone. FOLFOX, fluorouracil, leucovorin and oxaliplatin. BEACOPP, bleomycin, etoposide, doxorubicin, cyclophosphamide, vincristine, procarbazine and prednisolone. Euramos is a protocol for osteosarcoma consisting of methotrexate, cisplatin, doxorubicin and ifosfamide. EuroEWING12 is a protocol for Ewing sarcoma consisting of different combinations of vincristine, ifosfamide, doxorubicin, etoposide, actinomycin D and cyclophosphamide.

## References

[1] Ferrari A., Stark D., Peccatori F.A., Fern L., Laurence V., Gaspar N., Bozovic-Spasojevic I., Smith O., de Munter J., Derwich K. (2021). Adolescents and Young Adults (AYA) with Cancer: A Position Paper from the AYA Working Group of the European Society for Medical Oncology (ESMO) and the European Society for Paediatric Oncology (SIOPE). ESMO Open.

[2] Anderson B., Albritton K. (2006). Closing the Gap: Research and Care Imperatives for Adolescents and Young Adults with Cancer.

[3] Miller K.D., Fidler-Benaoudia M., Keegan T.H., Hipp H.S., Jemal A., Siegel R.L. (2020). Cancer Statistics for Adolescents and Young Adults, 2020. CA Cancer J. Clin..

[4] You L., Lv Z., Li C., Ye W., Zhou Y., Jin J., Han Q. (2021). Worldwide Cancer Statistics of Adolescents and Young Adults in 2019: A Systematic Analysis of the Global Burden of Disease Study 2019. ESMO Open.

[5] Sodergren S.C., Husson O., Robinson J., Rohde G.E., Tomaszewska I.M., Vivat B., Dyar R., Darlington A.S. (2017). Systematic Review of the Health-Related Quality of Life Issues Facing Adolescents and Young Adults with Cancer. Qual. Life Res..

[6] Husson O., Huijgens P.C., van der Graaf W.T.A. (2018). Psychosocial Challenges and Health-Related Quality of Life of Adolescents and Young Adults with Hematologic Malignancies. Blood.

[7] Anderson R.A., Amant F., Braat D., D’Angelo A., Chuva de Sousa Lopes S.M., Demeestere I., Dwek S., Frith L., Lambertini M., Maslin C. (2020). ESHRE Guideline: Female Fertility Preservation†. Hum. Reprod. Open.

[8] Oktay K., Harvey B.E., Partridge A.H., Quinn G.P., Reinecke J., Taylor H.S., Hamish Wallace W., Wang E.T., Loren A.W. (2018). Fertility Preservation in Patients with Cancer: ASCO Clinical Practice Guideline Update. J. Clin. Oncol..

[9] Lambertini M., Peccatori F.A., Demeestere I., Amant F., Wyns C., Stukenborg J.B., Paluch-Shimon S., Halaska M.J., Uzan C., Meissner J. (2020). Fertility Preservation and Post-Treatment Pregnancies in Post-Pubertal Cancer Patients: ESMO Clinical Practice Guidelines†. Ann. Oncol..

[10] Guida M., Antonietta Castaldi M., Rosamilio R., Giudice V., Orio F., Selleri C. (2016). Reproductive Issues in Patients Undergoing Hematopoietic Stem Cell Transplantation: An Update. J. Ovarian Res..

[11] Janssen S.H.M., van der Graaf W.T.A., van der Meer D.J., Manten-Horst E., Husson O. (2021). Adolescent and Young Adult (Aya) Cancer Survivorship Practices: An Overview. Cancers.

[12] Coccia P.F., Pappo A.S., Beaupin L., Borges V.F., Borinstein S.C., Chugh R., Dinner S., Folbrecht J., Frazier A.L., Goldsby R. (2018). Adolescent and Young Adult Oncology, Version 2.2018, NCCN Clinical Practice Guidelines in Oncology. J. Natl. Compr. Canc Netw..

[13] Santaballa A., Márquez-Vega C., Rodríguez-Lescure Á., Rovirosa Á., Vázquez L., Zeberio-Etxetxipia I., Andrés M., Bassas L., Ceballos-Garcia E., Domingo J. (2022). Multidisciplinary Consensus on the Criteria for Fertility Preservation in Cancer Patients. Clin. Transl. Oncol..

[14] Cosgrove C.M., Salani R. (2019). Ovarian Effects of Radiation and Cytotoxic Chemotherapy Damage. Best Pract. Res. Clin. Obs. Gynaecol..

[15] Sonigo C., Beau I., Binart N., Grynberg M. (2019). The Impact of Chemotherapy on the Ovaries: Molecular Aspects and the Prevention of Ovarian Damage. Int. J. Mol. Sci..

[16] Anderson R.A., Cameron D., Clatot F., Demeestere I., Lambertini M., Nelson S.M., Peccatori F. (2022). Anti-Müllerian Hormone as a Marker of Ovarian Reserve and Premature Ovarian Insufficiency in Children and Women with Cancer: A Systematic Review. Hum. Reprod. Update.

[17] Bedoschi G., Navarro P.A., Oktay K. (2016). Chemotherapy-Induced Damage to Ovary: Mechanisms and Clinical Impact. Future Oncol..

[18] Christian N., Gemignani M.L. (2019). Issues with Fertility in Young Women with Breast Cancer. Curr. Oncol. Rep..

[19] Miller J.J., Williams G.F., Leissring J.C. (1971). Multiple Late Complications of Therapy with Cyclophosphamide, Including Ovarian Destruction. Am. J. Med..

[20] Chow E.J., Stratton K.L., Leisenring W.M., Oeffinger K.C., Sklar C.A., Donaldson S.S., Ginsberg J.P., Kenney L.B., Levine J.M., Robison L.L. (2016). Pregnancy after Chemotherapy in Male and Female Survivors of Childhood Cancer Treated between 1970 and 1999: A Report from the Childhood Cancer Survivor Study Cohort. Lancet Oncol..

[21] Anderson R.A., Brewster D.H., Wood R., Nowell S., Fischbacher C., Kelsey T.W., Wallace W.H.B. (2018). The Impact of Cancer on Subsequent Chance of Pregnancy: A Population-Based Analysis. Hum. Reprod..

[22] Di Meglio A., Vaz-Luis I., Pistilli B., Di Meglio A., Vaz-Luis I., Pistilli B. (2020). Impact of Systemic Anticancer Therapy on Fertility. Fertility Challenges and Solutions in Women with Cancer.

[23] Dinas K.D. (2020). Impact of Breast Cancer Treatment on Fertility. Diseases of the Breast during Pregnancy and Lactation.

[24] Vo K.C.T., Kawamura K. (2021). Female Oncofertility: Current Understandings, Therapeutic Approaches, Controversies, and Future Perspectives. J. Clin. Med..

[25] Martelli V., Latocca M.M., Ruelle T., Perachino M., Arecco L., Beshiri K., Razeti M.G., Tagliamento M., Cosso M., Fregatti P. (2021). Comparing the Gonadotoxicity of Multiple Breast Cancer Regimens: Important Understanding for Managing Breast Cancer in Pre-Menopausal Women. Breast Cancer Targets Ther..

[26] Reddy N., Furness C.L., Davies M.C. (2018). Fertility in the Adolescent and Young Adult Patient with Cancer. A Practical Approach to the Care of Adolescents and Young Adults with Cancer.

[27] Chan J.L., Wang E.T. (2017). Oncofertility for Women with Gynecologic Malignancies. Gynecol. Oncol..

[28] Alesi L.R., Winship A.L., Hutt K.J. (2021). Evaluating the Impacts of Emerging Cancer Therapies on Ovarian Function. Curr. Opin. Endocr. Metab. Res..

[29] Szymanska K.J., Tan X., Oktay K. (2020). Unraveling the Mechanisms of Chemotherapy-Induced Damage to Human Primordial Follicle Reserve: Road to Developing Therapeutics for Fertility Preservation and Reversing Ovarian Aging. Mol. Hum. Reprod..

[30] Silva C., Cristina A., Rama R., Soares S.R., Moura-Ramos M., Almeida-Santos T. (2019). Adverse Reproductive Health Outcomes in a Cohort of Young Women with Breast Cancer Exposed to Systemic Treatments. J. Ovarian Res..

[31] Moftakhar B., Vitek W., Huston A. (2020). Impact of Breast Cancer Systemic Therapies on Fertility. Curr. Breast Cancer Rep..

[32] Lasica M., Taylor E., Bhattacharyya P., Bennett A., Cooke R.E., Stern C., Agresta F., Ayton R., Grigg A. (2016). Fertility in Premenopausal Women Post Autologous Stem Cell Transplant with BEAM Conditioning. Eur. J. Haematol..

[33] Overbeek A., van den Berg M.H., van Leeuwen F.E., Kaspers G.J.L., Lambalk C.B., van Dulmen-den Broeder E. (2017). Chemotherapy-Related Late Adverse Effects on Ovarian Function in Female Survivors of Childhood and Young Adult Cancer: A Systematic Review. Cancer Treat. Rev..

[34] Goodwin P.J., Ennis M., Pritchard K.I., Trudeau M., Hood N. (1999). Risk of Menopause during the First Year after Breast Cancer Diagnosis. J. Clin. Oncol..

[35] Loren A.W., Senapati S. (2019). Fertility Preservation in Patients with Hematologic Malignancies and Recipients of Hematopoietic Cell Transplants. Blood.

[36] Hoelder S., Clarke P.A., Workman P. (2012). Discovery of Small Molecule Cancer Drugs: Successes, Challenges and Opportunities. Mol. Oncol..

[37] Bussies P.L., Richards E.G., Rotz S.J., Falcone T. (2022). Targeted Cancer Treatment and Fertility: Effect of Immunotherapy and Small Molecule Inhibitors on Female Reproduction. Reprod. Biomed. Online.

[38] Bang Y.J., Van Cutsem E., Feyereislova A., Chung H.C., Shen L., Sawaki A., Lordick F., Ohtsu A., Omuro Y., Satoh T. (2010). Trastuzumab in Combination with Chemotherapy versus Chemotherapy Alone for Treatment of HER2-Positive Advanced Gastric or Gastro-Oesophageal Junction Cancer (ToGA): A Phase 3, Open-Label, Randomised Controlled Trial. Lancet.

[39] von Minckwitz G., Procter M., de Azambuja E., Zardavas D., Benyunes M., Viale G., Suter T., Arahmani A., Rouchet N., Clark E. (2017). Adjuvant Pertuzumab and Trastuzumab in Early HER2-Positive Breast Cancer. N. Engl. J. Med..

[40] Cameron D., Piccart-Gebhart M.J., Gelber R.D., Procter M., Goldhirsch A., de Azambuja E., Castro G., Untch M., Smith I., Gianni L. (2017). 11 Years’ Follow-up of Trastuzumab after Adjuvant Chemotherapy in HER2-Positive Early Breast Cancer: Final Analysis of the HERceptin Adjuvant (HERA) Trial. Lancet.

[41] Richani D., Gilchrist R.B. (2018). The Epidermal Growth Factor Network: Role in Oocyte Growth, Maturation and Developmental Competence. Hum. Reprod. Update.

[42] Rambhatla A., Strug M.R., De Paredes J.G., Cordoba Munoz M.I., Thakur M. (2021). Fertility Considerations in Targeted Biologic Therapy with Tyrosine Kinase Inhibitors: A Review. J. Assist. Reprod. Genet..

[43] Dosiou C. (2020). Thyroid and Fertility: Recent Advances. Thyroid.

[44] Robert C. (2020). A Decade of Immune-Checkpoint Inhibitors in Cancer Therapy. Nat. Commun..

[45] Schmid P., Cortes J., Dent R., Pusztai L., McArthur H., Kümmel S., Bergh J., Denkert C., Park Y.H., Hui R. (2022). Event-Free Survival with Pembrolizumab in Early Triple-Negative Breast Cancer. N. Engl. J. Med..

[46] Eggermont A.M.M., Blank C.U., Mandalà M., Long G.V., Atkinson V.G., Dalle S., Haydon A.M., Meshcheryakov A., Khattak A., Carlino M.S. (2021). Adjuvant Pembrolizumab versus Placebo in Resected Stage III Melanoma (EORTC 1325-MG/KEYNOTE-054): Distant Metastasis-Free Survival Results from a Double-Blind, Randomised, Controlled, Phase 3 Trial. Lancet Oncol..

[47] Ribas A., Wolchok J.D. (2018). Cancer Immunotherapy Using Checkpoint Blockade. Science.

[48] Topalian S.L., Hodi F.S., Brahmer J.R., Gettinger S.N., Smith D.C., McDermott D.F., Powderly J.D., Sosman J.A., Atkins M.B., Leming P.D. (2019). Five-Year Survival and Correlates Among Patients with Advanced Melanoma, Renal Cell Carcinoma, or Non-Small Cell Lung Cancer Treated with Nivolumab. JAMA Oncol..

[49] Robert C., Marabelle A., Herrscher H., Caramella C., Rouby P., Fizazi K., Besse B. (2020). Immunotherapy Discontinuation—How, and When? Data from Melanoma as a Paradigm. Nat. Rev. Clin. Oncol..

[50] Garutti M., Lambertini M., Puglisi F. (2021). Checkpoint Inhibitors, Fertility, Pregnancy, and Sexual Life: A Systematic Review. ESMO Open.

[51] Duma N., Lambertini M. (2020). It Is Time to Talk About Fertility and Immunotherapy. Oncologist.

[52] Tanda E.T., Croce E., Spagnolo F., Zullo L., Spinaci S., Genova C., Rossi G. (2021). Immunotherapy in Adolescents and Young Adults: What Remains in Cancer Survivors?. Front. Oncol..

[53] Postow M.A., Sidlow R., Hellmann M.D. (2018). Immune-Related Adverse Events Associated with Immune Checkpoint Blockade. N. Engl. J. Med..

[54] Traila A., Dima D., Achimas-Cadariu P., Micu R. (2018). Fertility Preservation in Hodgkin’s Lymphoma Patients That Undergo Targeted Molecular Therapies: An Important Step Forward from the Chemotherapy Era. Cancer Manag. Res..

[55] Berjeb K.K., Debbabi L., Braham M., Zemni Z., Chtourou S., Hannachi H., Hamdoun M., Ayadi M., Kacem K., Zhioua F. (2021). Evaluation of Ovarian Reserve before and after Chemotherapy. J. Gynecol. Obs. Hum. Reprod..

[56] Salama M., Woodruff T.K. (2017). Anticancer Treatments and Female Fertility: Clinical Concerns and Role of Oncologists in Oncofertility Practice. Expert Rev. Anticancer. Ther..

[57] Algarroba G.N., Sanfilippo J.S., Valli-Pulaski H. (2018). Female Fertility Preservation in the Pediatric and Adolescent Cancer Patient Population. Best. Pract. Res. Clin. Obs. Gynaecol..

[58] Balachandren N., Davies M. (2017). Fertility, Ovarian Reserve and Cancer. Maturitas.

[59] Oncofertility Consensus Document Compiled by Members of the CCLG Late Effects Group (2019). Subfertility Risk Consensus Document: Update 2008. cclg.org.uk.

[60] Lambertini M., Del Mastro L., Pescio M.C., Andersen C.Y., Azim H.A., Peccatori F.A., Costa M., Revelli A., Salvagno F., Gennari A. (2016). Cancer and Fertility Preservation: International Recommendations from an Expert Meeting. BMC Med..

[61] van Dorp W., Haupt R., Anderson R.A., Mulder R.L., van den Heuvel-Eibrink M.M., van Dulmen-den Broeder E., Su H.I., Winther J.F., Hudson M.M., Levine J.M. (2018). Reproductive Function and Outcomes in Female Survivors of Childhood, Adolescent, and Young Adult Cancer: A Review. J. Clin. Oncol..

[62] van den Berg M.H., van Dijk M., Byrne J., Berger C., Dirksen U., Winther J.F., Fossa S.D., Grabow D., Grandage V.L., Haupt R. (2021). Treatment-Related Fertility Impairment in Long-Term Female Childhood, Adolescent and Young Adult Cancer Survivors: Investigating Dose-Effect Relationships in a European Case-Control Study (PanCareLIFE). Hum. Reprod..

[63] Cardoso F., Kyriakides S., Ohno S., Penault-Llorca F., Poortmans P., Rubio I.T., Zackrisson S., Senkus E. (2019). Early Breast Cancer: ESMO Clinical Practice Guidelines for Diagnosis, Treatment and Follow-Up†. Ann. Oncol..

[64] Zhao J., Liu J., Chen K., Li S., Wang Y., Yang Y., Deng H., Jia W., Rao N., Liu Q. (2014). What Lies behind Chemotherapy-Induced Amenorrhea for Breast Cancer Patients: A Meta-Analysis. Breast Cancer Res. Treat..

[65] Bines J., Oleske D.M., Cobleigh M.A. (1996). Ovarian Function in Premenopausal Women Treated with Adjuvant Chemotherapy for Breast Cancer. J. Clin. Oncol..

[66] Goldhirsch A., Gelber R.D., Castiglione M. (1990). The Magnitude of Endocrine Effects of Adjuvant Chemotherapy for Premenopausal Breast Cancer Patients. The International Breast Cancer Study Group. Ann. Oncol..

[67] Parulekar W.R., Day A.G., Ottaway J.A., Shepherd L.E., Trudeau M.E., Bramwell V., Levine M., Pritchard K.I. (2005). Incidence and Prognostic Impact of Amenorrhea during Adjuvant Therapy in High-Risk Premenopausal Breast Cancer: Analysis of a National Cancer Institute of Canada Clinical Trials Group Study—NCIC CTG MA.5. J. Clin. Oncol..

[68] Ganz P.A., Land S.R., Geyer C.E., Cecchini R.S., Costantino J.P., Pajon E.R., Fehrenbacher L., Atkins J.N., Polikoff J.A., Vogel V.G. (2011). Menstrual History and Quality-of-Life Outcomes in Women with Node-Positive Breast Cancer Treated with Adjuvant Therapy on the NSABP B-30 Trial. J. Clin. Oncol..

[69] Elis A., Tevet A., Yerushalmi R., Blickstein D., Bairy O., Dann E., Blumenfeld Z., Abraham A., Manor Y., Shpilberg O. (2006). Fertility Status among Women Treated for Aggressive Non-Hodgkin’s Lymphoma. Leuk. Lymphoma.

[70] Behringer K., Mueller H., Goergen H., Thielen I., Eibl A.D., Stumpf V., Wessels C., Wiehlpütz M., Rosenbrock J., Halbsguth T. (2013). Gonadal Function and Fertility in Survivors after Hodgkin Lymphoma Treatment within the German Hodgkin Study Group HD13 to HD15 Trials. J. Clin. Oncol..

[71] Eichenauer D.A., Aleman B.M.P., André M., Federico M., Hutchings M., Illidge T., Engert A., Ladetto M. (2018). Hodgkin Lymphoma: ESMO Clinical Practice Guidelines for Diagnosis, Treatment and Follow-Up. Ann. Oncol..

[72] Budman D.R., Berry D.A., Cirrincione C.T., Henderson I.C., Wood W.C., Weiss R.B., Ferree C.R., Muss H.B., Green M.R., Norton L. (1998). Dose and Dose Intensity as Determinants of Outcome in the Adjuvant Treatment of Breast Cancer. J. Natl. Cancer Inst..

[73] Venturini M., Del Mastro L., Aitini E., Baldini E., Caroti C., Contu A., Testore F., Brema F., Pronzato P., Cavazzini G. (2005). Dose-Dense Adjuvant Chemotherapy in Early Breast Cancer Patients: Results from a Randomized Trial. J. Natl. Cancer Inst..

[74] Lambertini M., Ceppi M., Cognetti F., Cavazzini G., De Laurentiis M., De Placido S., Michelotti A., Bisagni G., Durando A., Valle E. (2017). Dose-Dense Adjuvant Chemotherapy in Premenopausal Breast Cancer Patients: A Pooled Analysis of the MIG1 and GIM2 Phase III Studies. Eur. J. Cancer.

[75] Engert A., Plütschow A., Eich H.T., Lohri A., Dörken B., Borchmann P., Berger B., Greil R., Willborn K.C., Wilhelm M. (2010). Reduced Treatment Intensity in Patients with Early-Stage Hodgkin’s Lymphoma. N. Engl. J. Med..

[76] Decanter C., Morschhauser F., Pigny P., Lefebvre C., Gallo C., Dewailly D. (2010). Anti-Müllerian Hormone Follow-up in Young Women Treated by Chemotherapy for Lymphoma: Preliminary Results. Reprod. Biomed. Online.

[77] Martin M., Pienkowski T., Mackey J., Pawlicki M., Guastalla J.-P., Weaver C., Tomiak E., Al-Tweigeri T., Chap L., Juhos E. (2005). Adjuvant Docetaxel for Node-Positive Breast Cancer. N. Engl. J. Med..

[78] Silva C., Caramelo O., Almeida-Santos T., Rama A.C.R. (2016). Factors Associated with Ovarian Function Recovery after Chemotherapy for Breast Cancer: A Systematic Review and Meta-Analysis. Hum. Reprod..

[79] Lambertini M., Campbell C., Bines J., Korde L.A., Izquierdo M., Fumagalli D., Del Mastro L., Ignatiadis M., Pritchard K., Wolff A.C. (2019). Adjuvant Anti-HER2 Therapy, Treatment-Related Amenorrhea, and Survival in Premenopausal HER2-Positive Early Breast Cancer Patients. JNCI J. Natl. Cancer Inst..

[80] Davis A.L., Klitus M., Mintzer D.M. (2005). Chemotherapy-Induced Amenorrhea from Adjuvant Breast Cancer Treatment: The Effect of the Addition of Taxanes. Clin. Breast Cancer.

[81] Tham Y.L., Sexton K., Weiss H., Elledge R., Friedman L.C., Kramer R. (2007). The Rates of Chemotherapy-Induced Amenorrhea in Patients Treated with Adjuvant Doxorubicin and Cyclophosphamide Followed by a Taxane. Am. J. Clin. Oncol..

[82] Gast K.C., Cathcart-Rake E.J., Norman A.D., Eshraghi L., Obidegwu N., Nichols H.B., Rosenberg S., Su H.I., Stewart E.A., Couch F.J. (2019). Regimen-Specific Rates of Chemotherapy-Related Amenorrhea in Breast Cancer Survivors. JNCI Cancer Spectr..

[83] Morarji K., McArdle O., Hui K., Gingras-Hill G., Ahmed S., Greenblatt E.M., Warner E., Sridhar S., Ali A.M.F., Azad A. (2017). Ovarian Function after Chemotherapy in Young Breast Cancer Survivors. Curr. Oncol..

[84] Ruddy K.J., Guo H., Barry W., Dang C.T., Yardley D.A., Moy B., Marcom P.K., Albain K.S., Rugo H.S., Ellis M.J. (2015). Chemotherapy-Related Amenorrhea after Adjuvant Paclitaxel-Trastuzumab (APT Trial). Breast Cancer Res. Treat..

[85] Lambertini M., Ceppi M., Anderson R.A., Cameron D.A., Bruzzone M., Franzoi M.A., Massarotti C., El-Abed S., Wang Y., Lecocq C. (2023). Impact of Anti-HER2 Therapy Alone and With Weekly Paclitaxel on the Ovarian Reserve of Young Women With HER2-Positive Breast Cancer. J. Natl. Compr. Cancer Netw..

[86] Ruddy K.J., Zheng Y., Tayob N., Hu J., Dang C.T., Yardley D.A., Isakoff S.J., Valero V.V., Faggen M.G., Mulvey T.M. (2021). Chemotherapy-Related Amenorrhea (CRA) after Adjuvant Ado-Trastuzumab Emtansine (T-DM1) Compared to Paclitaxel in Combination with Trastuzumab (TH) (TBCRC033: ATEMPT Trial). Breast Cancer Res. Treat..

[87] Lorenzi E., Simonelli M., Persico P., Dipasquale A., Santoro A. (2021). Risks of Molecular Targeted Therapies to Fertility and Safety during Pregnancy: A Review of Current Knowledge and Future Needs. Expert Opin. Drug Saf..

[88] Allegra C.J., Yothers G., O’Connell M.J., Sharif S., Petrelli N.J., Colangelo L.H., Atkins J.N., Seay T.E., Fehrenbacher L., Goldberg R.M. (2011). Phase III Trial Assessing Bevacizumab in Stages II and III Carcinoma of the Colon: Results of NSABP Protocol C-08. J. Clin. Oncol..

[89] Gharwan H., Lai C., Grant C., Dunleavy K., Steinberg S.M., Shovlin M., Fojo T., Wilson W.H. (2016). Female Fertility Following Dose-Adjusted EPOCH-R Chemotherapy in Primary Mediastinal B-Cell Lymphomas. Leuk. Lymphoma.

[90] Christopoulos C., Dimakopoulou V., Rotas E. (2008). Primary Ovarian Insufficiency Associated with Imatinib Therapy. N. Engl. J. Med..

[91] de Sanctis R., Lorenzi E., Agostinetto E., D’Amico T., Simonelli M., Santoro A. (2019). Primary Ovarian Insufficiency Associated with Pazopanib Therapy in a Breast Angiosarcoma Patient: A CARE-Compliant Case Report. Medicine.

[92] De Filette J., Andreescu C.E., Cools F., Bravenboer B., Velkeniers B. (2019). A Systematic Review and Meta-Analysis of Endocrine-Related Adverse Events Associated with Immune Checkpoint Inhibitors. Horm. Metab. Res..

[93] Duma N., Abdel-Ghani A., Yadav S., Hoversten K.P., Reed C.T., Sitek A.N., Enninga E.A.L., Paludo J., Aguilera J.V., Leventakos K. (2019). Sex Differences in Tolerability to Anti-Programmed Cell Death Protein 1 Therapy in Patients with Metastatic Melanoma and Non-Small Cell Lung Cancer: Are We All Equal?. Oncologist.

[94] Chatzidarellis E., Makrilia N., Giza L., Georgiadis E., Alamara C., Syrigos K.N. (2010). Effects of Taxane-Based Chemotherapy on Inhibin B and Gonadotropins as Biomarkers of Spermatogenesis. Fertil. Steril..

[95] Pallotti F., Pelloni M., Faja F., Di Chiano S., Di Rocco A., Lenzi A., Lombardo F., Paoli D. (2021). Semen Quality in Non-Hodgkin Lymphoma Survivors: A Monocentric Retrospective Study. Hum. Reprod..

[96] Wasilewski-Masker K., Seidel K.D., Leisenring W., Mertens A.C., Shnorhavorian M., Ritenour C.W., Stovall M., Green D.M., Sklar C.A., Armstrong G.T. (2014). Male Infertility in Long-Term Survivors of Pediatric Cancer: A Report from the Childhood Cancer Survivor Study. J. Cancer Surviv..

[97] Vakalopoulos I., Dimou P., Anagnostou I., Zeginiadou T. (2015). Impact of Cancer and Cancer Treatment on Male Fertility. Hormones.

[98] Delessard M., Saulnier J., Rives A., Dumont L., Rondanino C., Rives N. (2020). Exposure to Chemotherapy During Childhood or Adulthood and Consequences on Spermatogenesis and Male Fertility. Int. J. Mol. Sci..

[99] Ghafouri-Fard S., Shoorei H., Abak A., Seify M., Mohaqiq M., Keshmir F., Taheri M., Ayatollahi S.A. (2021). Effects of Chemotherapeutic Agents on Male Germ Cells and Possible Ameliorating Impact of Antioxidants. Biomed. Pharmacother..

[100] Bahadur G., Ozturk O., Muneer A., Wafa R., Ashraf A., Jaman N., Patel S., Oyede A.W., Ralph D.J. (2005). Semen Quality before and after Gonadotoxic Treatment. Hum. Reprod..

[101] Meistrich M.L., Wilson G., Brown B.W., Da Cunha M.F., Lipshultz L.I. (1992). Impact of Cyclophosphamide on Long-Term Reduction in Sperm Count in Men Treated with Combination Chemotherapy for Ewing and Soft Tissue Sarcomas. Cancer.

[102] Pryzant R.M., Meistrich M.L., Wilson G., Brown B., McLaughlin P. (1993). Long-Term Reduction in Sperm Count after Chemotherapy with and without Radiation Therapy for Non-Hodgkin’s Lymphomas. J. Clin. Oncol..

[103] Amin A., Brunckhorst O., Scott C., Wrench D., Gleeson M., Kazmi M., Ahmed K. (2021). ABVD and BEACOPP Regimens’ Effects on Fertility in Young Males with Hodgkin Lymphoma. Clin. Transl. Oncol..

[104] Van Der Kaaij M.A.E., Heutte N., Van Echten-Arends J., Raemaekers J.M.M., Carde P., Noordijk E.M., Fermé C., Thomas J., Eghbali H., Brice P. (2009). Sperm Quality before Treatment in Patients with Early Stage Hodgkin’s Lymphoma Enrolled in EORTC-GELA Lymphoma Group Trials. Haematologica.

[105] Bujan L., Walschaerts M., Moinard N., Hennebicq S., Saias J., Brugnon F., Auger J., Berthaut I., Szerman E., Daudin M. (2013). Impact of Chemotherapy and Radiotherapy for Testicular Germ Cell Tumors on Spermatogenesis and Sperm DNA: A Multicenter Prospective Study from the CECOS Network. Fertil. Steril..

[106] Lampe H., Horwich A., Norman A., Nicholls J., Dearnaley D.P. (1997). Fertility after Chemotherapy for Testicular Germ Cell Cancers. J. Clin. Oncol..

[107] Viviani S., Santoro A., Ragni G., Bonfante V., Bestetti O., Bonadonna G. (1985). Gonadal Toxicity after Combination Chemotherapy for Hodgkin’s Disease. Comparative Results of MOPP vs ABVD. Eur. J. Cancer Clin. Oncol..

[108] Chang X., Zhou L., Chen X., Xu B., Cheng Y., Sun S., Fang M., Xiang Y. (2017). Impact of Imatinib on the Fertility of Male Patients with Chronic Myelogenous Leukaemia in the Chronic Phase. Target. Oncol..

[109] Gambacorti-Passerini C., Tornaghi L., Cavagnini F., Rossi P., Pecori-Giraldi F., Mariani L., Cambiaghi N., Pogliani E., Corneo G., Gnessi L. (2003). Gynaecomastia in Men with Chronic Myeloid Leukaemia after Imatinib. Lancet.

[110] Brunet-Possenti F., Opsomer M.A., Gomez L., Ouzaid I., Descamps V. (2017). Immune Checkpoint Inhibitors-Related Orchitis. Ann. Oncol..

[111] Quach H.T., Robbins C.J., Balko J.M., Chiu C.Y., Miller S., Wilson M.R., Nelson G.E., Johnson D.B. (2019). Severe Epididymo-Orchitis and Encephalitis Complicating Anti-PD-1 Therapy. Oncologist.

[112] Scovell J.M., Benz K., Samarska I., Kohn T.P., Hooper J.E., Matoso A., Herati A.S. (2020). Association of Impaired Spermatogenesis with the Use of Immune Checkpoint Inhibitors in Patients with Metastatic Melanoma. JAMA Oncol..

[113] Salzmann M., Tosev G., Heck M., Schadendorf D., Maatouk I., Enk A.H., Hartmann M., Hassel J.C. (2021). Male Fertility during and after Immune Checkpoint Inhibitor Therapy: A Cross-Sectional Pilot Study. Eur. J. Cancer.

[114] Peters M., Pearlman A., Terry W., Mott S.L., Monga V. (2021). Testosterone Deficiency in Men Receiving Immunotherapy for Malignant Melanoma. Oncotarget.

[115] Rabinowitz M.J., Kohn T.P., Peña V.N., Samarska I.v., Matoso A., Herati A.S. (2020). Onset of Azoospermia in Man Treated with Ipilimumab/Nivolumab for BRAF Negative Metastatic Melanoma. Urol. Case Rep..

[116] Davies A., Naderpoor N., Parakh S. (2019). Isolated Hypogonadotropic Hypogonadism Secondary to Anti-Programmed Death Ligand 1 Inhibitor. J. Thorac. Oncol..

[117] Tulchiner G., Pichler R., Ulmer H., Staudacher N., Lindner A.K., Brunner A., Zelger B., Steinkohl F., Aigner F., Horninger W. (2021). Sex-Specific Hormone Changes during Immunotherapy and Its Influence on Survival in Metastatic Renal Cell Carcinoma. Cancer Immunol. Immunother..

[118] Practice Committee of the American Society for Reproductive Medicine (2019). Fertility Preservation in Patients Undergoing Gonadotoxic Therapy or Gonadectomy: A Committee Opinion. Fertil. Steril..

[119] Lambertini M., Horicks F., del Mastro L., Partridge A.H., Demeestere I. (2019). Ovarian Protection with Gonadotropin-Releasing Hormone Agonists during Chemotherapy in Cancer Patients: From Biological Evidence to Clinical Application. Cancer Treat. Rev..

[120] Spears N., Lopes F., Stefansdottir A., Rossi V., De Felici M., Anderson R.A., Klinger F.G. (2019). Ovarian Damage from Chemotherapy and Current Approaches to Its Protection. Hum. Reprod. Update.

[121] Morgan S., Lopes F., Gourley C., Anderson R.A., Spears N. (2013). Cisplatin and Doxorubicin Induce Distinct Mechanisms of Ovarian Follicle Loss; Imatinib Provides Selective Protection Only against Cisplatin. PLoS ONE.

[122] Oktay K., Turan V., Titus S., Stobezki R., Liu L. (2015). BRCA Mutations, DNA Repair Deficiency, and Ovarian Aging. Biol. Reprod..

